# Framing of and Attention to COVID-19 on Twitter: Thematic Analysis of Hashtags

**DOI:** 10.2196/30800

**Published:** 2021-09-10

**Authors:** Iman Tahamtan, Devendra Potnis, Ehsan Mohammadi, Laura E Miller, Vandana Singh

**Affiliations:** 1 School of Information Sciences The University of Tennessee Knoxville, TN United States; 2 School of Information Science The University of South Carolina Columbia, SC United States; 3 School of Communication Studies The University of Tennessee Knoxville, TN United States

**Keywords:** COVID-19, framing, Twitter, social media, public opinion, engagement, public attention, thematic analysis, public health

## Abstract

**Background:**

Although past research has focused on COVID-19–related frames in the news media, such research may not accurately capture and represent the perspectives of people from diverse backgrounds. Additionally, research on the public attention to COVID-19 as reflected through frames on social media is scarce.

**Objective:**

This study identified the frames about the COVID-19 pandemic in the public discourse on Twitter, which voices diverse opinions. This study also investigated the amount of public attention to those frames on Twitter.

**Methods:**

We collected 22 trending hashtags related to COVID-19 in the United States and 694,582 tweets written in English containing these hashtags in March 2020 and analyzed them via thematic analysis. Public attention to these frames was measured by evaluating the amount of public engagement with frames and public adoption of those frames.

**Results:**

We identified 9 frames including “public health guidelines,” “quarantine life,” “solidarity,” “evidence and facts,” “call for action,” “politics,” “post-pandemic life,” “shortage panic,” and “conflict.” Results showed that some frames such as “call for action” are more appealing than others during a global pandemic, receiving greater public adoption and engagement. The “call for action” frame had the highest engagement score, followed by “conflict” and “evidence and facts.” Additionally, “post-pandemic life” had the highest adoption score, followed by “call for action” and “shortage panic.” The findings indicated that the frequency of a frame on social media does not necessarily mean greater public adoption of or engagement with the frame.

**Conclusions:**

This study contributes to framing theory and research by demonstrating how trending hashtags can be used as new user-generated data to identify frames on social media. This study concludes that the identified frames such as “quarantine life” and “conflict” and themes such as “isolation” and “toilet paper panic” represent the consequences of the COVID-19 pandemic. The consequences could be (1) exclusively related to COVID-19, such as hand hygiene or isolation; (2) related to any health crisis such as social support of vulnerable groups; and (3) generic that are irrespective of COVID-19, such as homeschooling or remote working.

## Introduction

### Public Opinion on Twitter

COVID-19 is a global public health pandemic threatening millions of lives worldwide, leading to approximately 188,655,968 confirmed cases and 4,067,517 deaths across the globe as of July 16, 2021 [1]. Twitter was used as one of the major platforms for disseminating information and knowledge about the COVID-19 pandemic, resulting in massive data generated by the public about various aspects of the virus [2]. Twitter has become the most frequently used communication medium for disseminating health information since the outbreak of H1N1 in 2009 [3] and later during the outbreak of the H7N9 virus, or bird flu, in 2013 [4]. It was also a platform for discussing the Zika virus epidemic in 2015 and 2016 [5].

People use Twitter and other social media platforms to interact with, share their opinion about, and engage with public health messages in real time. Twitter allows public health gatekeepers to interact with the public directly. For instance, the Centers for Disease Control and Prevention, the World Health Organization (WHO), health care officials, and organizations used Twitter regularly to share public health messages about the pandemic and communicate its risks to the public [2].

The data shared on Twitter can be used to analyze and study public opinion. Understanding public opinion could help researchers and authorities identify the public’s needs, priorities, preferences, and behavior in real time. In turn, these data could impact public policy by encouraging governments and health care officials to distribute proper resources, take actions, and plan accordingly [6-8]. For instance, Avery [9] reported that monitoring public opinion about the Zika virus crisis on social media helped public information officers have a higher level of preparedness for managing the crisis.

### Frame Analysis of Public Opinion

Public opinion refers to people’s collective opinion about an issue such as COVID-19. The public consists of all the groups and subgroups in society, such as workers, doctors, officials, politicians, journalists, and students [10]. An effective strategy to understanding public opinion and attention to an issue is analyzing how people perceive and frame the issue [11,12]. Framing refers to selecting some aspects of an issue, promoting them, and making them salient [13]. Journalists and news media often use framing to conceptualize an issue, bring public attention to some aspects of the issue, and minimize attention to other aspects of it [14,15]. People also use framing to make sense of complex information, interpret and organize these ideas into comprehensible concepts, and present them to others [16].

Analyzing public opinion by frame analysis provides insights into the content and sentiment-based aspects of an issue [17]. Frames can serve as a starting point for designing effective messages to address people’s needs and concerns during public health crises [18]. Journalists, policymakers, professionals, and scientists use frames to communicate their messages in a more effective way that is easily understandable by the public [19]. Frames can also be used by governments, advocacy groups, and authorities to design public education materials, present more engaging and effective public dialogue, write more relevant stories for the public, and expand their audience, reach, and impact [20]. For instance, framing of messages related to COVID-19 can be used to design and target messages in an effective way to enhance public engagement with public health guidelines [21].

### Effect of Frames on Public Attention

An important aspect of framing research is to study the effect of frames on public attention. “Public attention refers to the general acknowledgement of a subject in the public sphere and subsequent civic discourse on the subject” [22]. Different frames about an issue can cause varied effects on public attention. A small change in how a topic is presented can sometimes cause a butterfly effect on public attention [15]. Framing or reframing an issue can shift how people understand the story, consequently changing how people respond to it [16]. Once the public turns its attention towards a series of frames about an issue and adopts those frames, it is likely that they collectively agree on the best decision and course of action [15]. The study by Krishnamurthy et al [23] showed that, when discussing the performance of medical treatments, the messages that are framed positively (ie, the chance of treatment success rather than its failure) had a greater impact on health treatment decisions. Almashat et al [24] indicated that survival frames (ie, the likelihood of surviving a certain procedure) would lead to making more informed medical decisions than mortality frames (ie, the possibility of dying from a certain procedure). Analyzing the effect of COVID-19 frames on public attention can be used to determine which frames are more effective in receiving greater public attention and impacting public opinion.

In summary, Twitter provides valuable user-generated data that can be beneficial for different stakeholders to respond to health crises. Although some studies have analyzed the frames about COVID-19 with a focus on the news media of politicians [18,25-28], there is no systematic study about the frames in public discourse on Twitter for this global pandemic. Therefore, this study aimed to fill this gap with the following research objectives.

### Research Objectives

The aim of this research was to (1) identify the frames associated with the COVID-19 pandemic in public discourse on Twitter and (2) analyze which of the COVID-19 frames in public discourse have received greater public attention on Twitter.

### Literature Review

#### Framing of the COVID-19 Pandemic

During public health emergencies, it is critical to communicate public health messages and guidelines to the public effectively. One way to make public health messages more effective is to frame them in a way that helps people understand health crises and positively impact their decision making and behavior. Previous empirical research shows that the news media play an important role in framing public health crises [25]. As evident in Multimedia Appendix 1, Ogbodo et al [26] analyzed the frames in 8 leading global media outlets globally, such as the BBC, News York Times, CNN, and People’s Daily. Poirier et al [27] investigated the frames used on the front pages of 12 well-known Canadian news media sources. Park et al [18] analyzed Korean COVID-19–related tweets to identify medical and nonmedical news frames, and Yu et al [25] studied the frames in the tweets of 2 major newspapers in Spain: El Mundo and El País.

The literature on the framing of COVID-19 shows that most past studies have focused on how COVID-19 has been framed by political figures and the news media [28]. These studies reflect the frames promoted by established gatekeepers, namely the news media, in society who may have some hidden agenda. Unlike previous framing studies, this study attempts to fill this gap by focusing on the frames related to COVID-19 in public discourse on Twitter, which voices the opinions of diverse groups of people.

#### Hashtags for Framing Messages on Twitter

Framing studies uses the features of communicating text to identify frames. The linguistic features include headlines or subheads of newspapers; photographs and photo captions; paragraphs of articles [29]; social media posts, such as tweets [30]; and other visual images, like icons [31]. For instance, Hellmueller and Zhang [32] used photographs to identify frames regarding the European refugee crisis in CNN International and Der Spiegel online news sites. Benziman [28] used US President and British Prime Minister speeches to identify how they framed issues related to COVID-19.

Hashtags are a linguistic feature that can be used to identify frames on Twitter and other social media platforms. Hashtags serve as catchphrases that can identify frames in tweets [33]. They represent a shared meaning or context [34] that, similar to frames, can highlight the most salient aspects of an issue (see [35]) in such a way as to promote it (see [13]).

In such circumstances as breaking or emerging events, a specific hashtag (eg, #BlackLivesMatter) or a set of hashtags (eg, #StayHome, #WashYourHands) become the main channel to represent an issue in online conversations by social media users. Hashtags that gain community-wide adoption and popularity can be used to identify the ad hoc framing of an event. Widely adopted hashtags show how the public frames a topic and can be used to determine the thematic frames of the issue [17]. Nevertheless, limited studies have acknowledged the possibility of using hashtags to identify frames (eg, [17,33]). Hence, this research explored hashtags in frame analysis as a new approach for framing theory and research.

#### Trending Hashtags in Frame Analysis

Among the numerous widely adopted hashtags on social media, some may become trendy and viral. Popular hashtags emerge in response to breaking news and other unexpected events, such as when an important, nationwide, or global issue happens [36,37].

Hashtags may go viral on Twitter as more and more people begin to use them [38]. Many users engage with trending topics and hashtags on social media [36]. Trending hashtags or topics often do not last long on Twitter [39]; however, they receive an initial increase of public attention, and then the focus of the public shifts elsewhere [34]. Trending topics and hashtags represent which issues have drawn the most public attention [36]. Trending hashtags also represent a community of online users who attend a unique topic or event for a limited period [34]. The mechanisms by which Twitter identifies the top trending topics and hashtags [36] or what causes some topics to become widely popular are not clear [39]. Nonetheless, such popularity peaks are of great relevance for identifying the issues that are the focal point of the public. As such, trending hashtags are suitable tools to be used in frame analysis of social media posts.

#### Benefits of Using Hashtags in Frame Analysis

Using hashtags in frame analysis has 2 benefits: It facilitates framing public opinion on social media, and it mitigates the subjectivity issue in frame analysis. Hashtags, similar to photographs [40], make it easy for researchers to understand what the tweet's content is about because they are the commonly accepted public signals for framing and presenting an event or topic among all social media platforms. While framing research on social media has focused mostly on analyzing social media posts, the question of how issues are framed through hashtags has remained relatively underexamined.

Content analysis is a dominant method for analyzing and identifying frames on social media (eg, [41]) and in non-social media contexts (eg, [42]). Matthes and Kohring [43] noted that a major issue with identifying frames through content analysis is that it involves researchers’ subjective biases in analyzing and coding the text differently. Researchers’ subjective biases question the validity and reliability of content analysis and the results in frame analysis [43]. Subjectivity is nearly impossible to avoid [19]; however, using hashtags to identify frames mitigates the subjective role of the researcher in frame analysis because hashtags have a “classificatory function” of indicating what a social media post is about [44]. Therefore, hashtags enhance the researcher’s capability to describe online information [45] and identify “textual aboutness” [46] of social media posts. In some cases, hashtags may not stand alone in understanding textual aboutness because textual aboutness can be sensitive to context. For instance, a hashtag might reflect 2 different meanings simultaneously [44].

#### The Effect of Frames on Public Attention on Social Media

There is no standard way for measuring public attention on social media [47]. However, some previous studies have used tweet activity, such as the retweet frequency, to measure public attention [47,48]. Ripberger et al [48] studied public attention by assessing the number of tornado-related tweets posted on Twitter. Ripberger et al [48] assumed that the increase in the number of tornado warnings and number of tornado watches (issued by 2 different institutes) led to an increase in the number of tweets (representing public attention). Chew and Eysenbach [3] also indicated a coincidence between major H1N1 news and the frequency of H1N1 tweets in April 2009. The presence of a relationship between tweet activity and public attention has been shown in other studies about swine flu [49] or influenza [50].

None of the framing studies on COVID-19 [18,21,25-28] have identified the effect of frames on public attention. This study investigated the effect of COVID-19 frames in public discourse on Twitter on public attention. This study took a new approach that measures public attention more accurately than the methods used in previous studies because it measured not only tweet activity (ie, number of likes and retweets a frame receives) but also how many unique Twitter users have adopted a frame. In this new approach, public attention was measured by evaluating the score of public engagement with frames and the score of public adoption of frames (see Methods, Measuring Public Attention). Public engagement refers to the number of likes and retweets each frame has received. Public adoption is assessed by the number of unique users in each frame category [51].

## Methods

### Data Collection

Twitter was the primary source of data collection (ie, hashtags and tweets) in this study. The *get_trend* function from the *rtweet* package in the R software was used to collect trending hashtags related to COVID-19 on Twitter in March 2020. Additionally, the trending feature on the Twitter website was observed at least 3 times a day for the entire month of March 2020 to track and record trending hashtags. The trending hashtags identified by the *get_trend* function were exactly similar to those identified on Twitter. In March 2020, 22 trending hashtags related to COVID-19 were identified on Twitter. Additionally, the trending hashtags from each day were searched in the search box of the Twitter website to find other relevant hashtags and achieve a greater degree of reliability in data collection. For instance, by searching #QuarantineLife, a trending hashtag on March 16, 2020, additional hashtags, including #QuarantineDogs and #QuarantineCats, were identified.

This study then collected the tweets that contained at least one of the trending or associated hashtags. The *search_tweets* function in the *rtweet* package was used to collect tweets by the R software and the streaming application programming interface (API). R, Salesforce Social Studio, and Brandwatch were used to collect 694,582 tweets written in English from March 1, 2020 to March 31, 2020.

This study removed the retweets in the dataset to analyze initial tweets posted by people on Twitter. Additionally, quote tweets (ie, the retweets with comments), representing the original comments posted by Twitter users, were analyzed.

### Bot Account Removal

Bots are automated accounts that can manipulate and impact public attention on social media [52]. Ferrara [52] indicated that bots were active during the COVID-19 pandemic and found that hashtags like #bitcoin, #smartnew, and #grreatwakening were mostly posted by bot accounts on Twitter. This study removed 269,854 (269,854/694,582, 38.85%) tweets posted by bot accounts to increase the validity of results, leaving 424,728 (424,728/694,582, 61.15%) tweets in the dataset.

The default model of the *tweetbotornot* package in R software was used to remove bot accounts [53]. The default model is 93.53% accurate in classifying bots and 95.32% accurate in classifying nonbots. It uses user-level features (eg, bio, location, number of followers and friends) and tweet-level features (eg, number of hashtags, mentions, capital letters) of Twitter accounts to identify bot accounts [53]. Accounts that received a score of at least 50% or a probability of 0.5 were considered bots and were removed [54].

### Thematic Analysis to Identify Frames

Hashtags were the main unit of analysis in this research and the building blocks of identifying frames. The frames identified in this study are primarily informed by the classification of trending and associated hashtags to different categories. Additionally, to identify the frames about COVID-19 in the public discourse on Twitter, thematic analysis was used. Thematic analysis is a method that provides a detailed description of the textual data [55]. In this research, public opinion includes all Twitter users such as workers, doctors, officials, journalists, politicians, teachers, and any other group on Twitter.

In thematic analysis, the judgment of the researcher or analyst is adequate for identifying themes [55]. The researcher analyzes the text and identifies themes within the “surface meanings of the data” without looking for anything beyond the text [55].

The thematic analysis helped us provide a more detailed and nuanced description of hashtags and find repeated patterns of meaning [55]. The contents of up to 100 randomly selected tweets were checked to ensure alignment between the hashtags and the contents of tweets. For instance, #WashYourHands, #HandWashing, #SafeHand, #SafeHands, and #HandWashChallenge were classified as one category named “hand hygiene.” Using thematic analysis, this study analyzed the content of the tweets associated with these hashtags to ensure they were related to hand hygiene. The thematic analysis also helped provide a more accurate description of this category. In some cases, the thematic analysis helped us redefine the theme assigned with a set of grouped hashtags.

**Figure 1:**
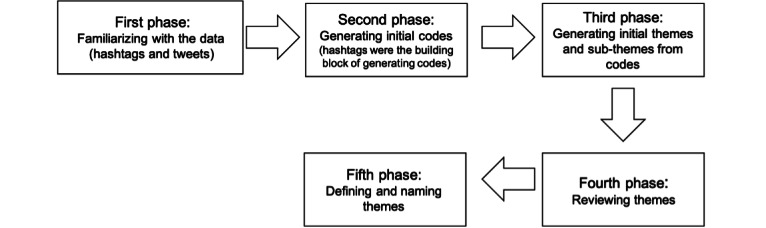
Workflow visualization for the 5 phases of the thematic analysis.

We followed 5 phases to analyze the themes in the tweets. Figure 1 presents the workflow visualization for the 5 phases of the thematic analysis. In the first phase of thematic analysis, researchers familiarized themselves with the data [56] by reading the hashtags and the tweets associated with them several times before coding. It helped researchers obtain some knowledge of the hashtags, related tweets, and the initial list of ideas and themes.

In the second phase, initial codes were generated from trending and associated hashtags [56]. Hashtags were used to describe what a particular tweet was about and to create codes. For instance, #ToiletPaperPanic is a hashtag in the data referring to the public’s panic for the possible shortage of toilet paper during the early days of the COVID-19 pandemic. Additionally, #WashYourHands is a hashtag that emphasizes the importance of hand hygiene to prevent contracting the COVID-19 virus.

In the third phase, codes were analyzed and classified into potential themes and subthemes [56]. For instance, the following set of hashtags — #SocialDistancing, #SocialDistancing, #KeepYourDistance, #PhysicalDistancing, #SocialDistance, #SocialDistancingNow, #YouAreTooCloseIf, #Dont BeASpreader, and #StoptheSpread — and the tweets associated with them helped the researchers to identify and propose the “social distancing” theme.

In phase 4, the researchers reviewed the themes and, when necessary, combined, refined, or separated them [56] to generate the final themes. In the fifth phase, the themes were clustered into frames. Concise names were assigned to the themes to help readers comprehend the theme’s meaning [56].

### Measuring Public Attention

This study used the scores of public engagement with and public adoption of frames to measure public attention to frames on Twitter. Following DeMasi et al [51], this study defined public engagement as the extent to which users engage with a frame. Public engagement was measured by the number of times a given frame had received at least one like or retweet divided by the frequency of that frame in the entire dataset. The lowest public engagement score that each frame could receive was 0, with a maximum of 1.

Public adoption refers to how broadly Twitter users adopt a frame. High adoption indicates a diverse community with many unique users who have posted tweets with hashtags in each frame. Low diversity shows a tight community with only a few users posting tweets with the hashtags in a given frame multiple times [51]. Public adoption was measured by the number of unique Twitter user accounts in a given frame divided by the frequency of the frame [51]. The public adoption score ranges from 0 to 1 as the minimum and maximum scores, respectively.

## Results

### Framing of COVID-19 on Twitter

We identified 9 categories of frames through thematic analysis (see Multimedia Appendix 2). Each frame is defined by a set of consequences of COVID-19 along with hashtags, characterizing what each frame category describes. The frames are “public health guidelines,” “quarantine life,” “solidarity,” “evidence and facts,” “call for action,” “politics,” “post-pandemic life,” “shortage panic,” and “conflict.”

#### Public Health Guidelines

Public health guidelines consisted of 5 main themes: isolation, social distancing, hand hygiene, face hygiene, preventive tips, and awareness. Isolation included hashtags that encourage and advise people to isolate themselves to prevent the spread of the virus. Some of the hashtags in this theme, such as #StayHomeStayHealthy or #StayHomeSaveLives, positively motivate people to self-isolate by staying home. This frame also included the recommendations people made to call others’ attention to the precautionary measures required to reduce the chance of being infected by the virus, such as practicing social distancing and face and hand hygiene (eg, #SocialDistancing, #WashYourHands, and #WearAMask). Sometimes tweets contained hashtags with a sense of humor and positive sentiments, such as #YouAreTooCloseIf or #How toKeepPeopleHome.

Other hashtags in this frame, such as #CoronaVirusTips, were used to provide guidelines and tips about various aspects of the virus, including how to clean smartphones, wash hands, and do contactless delivery of food. Additionally, this frame included tips for preventing potential exposure to the virus. For instance, #CoronaVirusPrevention was used to show how police departments should prevent potential disclosure and provide coronavirus prevention tips for people with disabilities.

#### Quarantine Life

This frame contained several themes that focused on people’s daily lives during the quarantine. People used various hashtags to share stories, pictures, or videos of their home offices, the homeschooling of kids, and their pets. Pet owners used hashtags such as #QuarantineCats or #QuarantineDogs to share humorous content about their living experiences in isolation. #After3WeeksWithMyFamily and #SideEffectsofQuarantineLife were used to post amusing messages along with photos or videos, often to make fun of the lived experience and actual difficulties people were dealing with during the pandemic. Quarantine life also focused on the impact of the pandemic on how people worked and learned during the quarantine. People posted videos or pictures of themselves using hashtags such as #RemoteLearning, #DistanceLearning, #RemoteWorking, or #OnlineLearning while working or learning from home.

#### Solidarity

Solidarity focused on inspiring, encouraging, and giving hope to each other; social support; providing voluntary services to communities; acknowledging health care professionals; and emphasizing unity. People used hashtags such as #StayStrong or #TogetherApart to inspire “staying home together” during the pandemic. The hashtags #CoronaWarriors, #ClapForCarers, #HealthCareHeroes, and #ClapForOurCarers were used to show appreciation to the frontline workers in fighting the pandemic, namely health care professionals.

A major theme in solidarity was social support, which consisted of hashtags used to assist various (vulnerable) populations or services. For instance, #ProtectOurSeniors was used to emphasize the importance of supporting seniors medically, financially, and emotionally. Other hashtags such as #OpenForTakeout, #OpenForDelivery, #SupportSmallBusiness, and #SupportLocal were used to support businesses and acknowledge the stores open for takeout or delivery during the pandemic.

#### Call for Action

This frame refers to a lack of accountability and responsibility in governments for not taking necessary actions to close public places. This frame also consisted of hashtags that show that the public is also responsible for their lack of compliance with public health guidelines. For instance, #CloseTheBeaches was used in tweets to emphasize that a public space like beaches should be closed as many people went to Florida’s beaches during the pandemic.

#### Evidence and Facts

This category focused on updates, reality, and truths about various aspects of the COVID-19 virus, such as the total number of deaths, new cases, discharged patients, and further evidence about how the virus spreads. For instance, #CoronaVirusUpdates was used on March 29, 2020 to refer to emerging hotspots of coronavirus and emphasized that New York remained the worst-hit US state. Another hashtag in this category was #CoronaVirusTruth, which was used to provide people with the facts and truths about COVID-19, such as the increasing number of deaths across the globe.

#### Politics

This frame refers to the accountability of politicians for their national policies and actions during the pandemic. Some hashtags were used to hold political officials accountable for their policies, decisions, and actions regarding the COVID-19 pandemic. The tweets in this category often had a negative sentiment. For instance, #TrumpLiedPeopleDied was used to show a lack of transparency and timely action to control the virus, not taking the pandemic seriously, and not following public health guidelines and policies. #ChinaLiedPeopleDied was, on the other hand, used to make China responsible for the transmission of the COVID-19 virus to other countries.

#### Post-Pandemic Life

This frame focused on the positive sentiment surrounding people’s plans, feelings, and life after the pandemic is over. #WhenCoronaVirusIsOver and #WhenILeaveMyHouseAgain are the hashtags in this category that sometimes were posted with tweets with a sense of humor. For example, a tweet stated, “people are going to stay home even when the pandemic is over.” People used the hashtags in this category to refer to the food they like to eat; to describe the places or people they enjoy visiting; to recommend things people should avoid, like eating animals; to emphasize that the world would be a cleaner place after the pandemic; and to suggest that people would love and respect nature more than in the past.

#### Shortage Panic

This frame showed panic about the shortage of products as a top priority of people when the pandemic had just started in March 2020. Shortage panic refers to the public reaction to and anxiety about the shortage of resources, including bottled water, toilet paper, hand sanitizer, and food. Most tweets in this category contained an image showing empty shelves in stores or people lined up in stores trying to purchase products such as toilet paper.

This frame demonstrated uncertainty in the public about how much supplies were needed because the future was unpredictable [57]. The uncertainty about the pandemic led to an “exaggerated sense of urgency and a fear of scarcity,” which resulted in actual scarcity [57].

#### Conflict

Conflicts refers to arguments and disagreements among people. Hashtags such as #FilmYourHospital or #EmptyHospitals were used to imply that COVID-19 was not as severe as stated by the media, governments, or health officials. Some people used these hashtags to frame their disapproval of the lockdown and social distancing or to suggest that COVID-19 is not real by filming empty hospitals. Another hashtag in this category was #FakeNews, which people used to share opposing views about various aspects of COVID-19. It was also used to show disapproval with the information and news spread about the virus, mostly by the news media or politicians from both the Democratic and Republican parties. #FakeNews was sometimes used to argue in favor of issues related to COVID-19, and other times, it was used to argue against those issues (see [19]).

### Public Attention to Frames

Public attention to frames was assessed using the 2 measures of “public engagement” and “public adoption.” Public engagement shows how engaging a frame has been on Twitter, counted by the number of retweets and likes a frame had received. Public adoption refers to the number of unique Twitter user accounts in a given frame category, divided by the frequency of frames in that frame category [58].

Table 1 shows public engagement with each frame. “Call for action” had the highest engagement (0.68), followed by “conflict” (0.67) and “evidence and facts” (0.59). Table 2 shows the public adoption score for each frame. “Post-pandemic life” (0.99) had the highest adoption score, followed by “call for action” (0.92) and “shortage panic” (0.91).

**Table 1:** Public engagement with frames.

Frame number	Frame	Frequency of the frame, n	Labeled Yes^a^, n	Labeled No^a^, n	Public engagement score (Yes/Yes+No)
1	Public health guidelines	171,009	94,545	76,464	0.55
2	Quarantine life	86,983	48,701	38,282	0.56
3	Solidarity	46,164	25,253	20,911	0.54
4	Evidence and facts	31,819	18,874	12,945	0.59
5	Call for action	31,809	21,881	9928	0.68
6	Politics	24,346	11,903	12,443	0.49
7	Post-pandemic life	20,442	11,439	9003	0.56
8	Shortage panic	16,815	9321	7494	0.55
9	Conflict	3550	2384	1166	0.67

^a^Each frame receiving at least a retweet or like was labeled as “Yes”; otherwise, it was labeled as “No.”

**Table 2:** Public adoption of frames.

Frame number	Frame	Frequency of unique users (U), n	Frequency of frame (F), n	Public adoption score (U/F)
1	Public health guidelines	142,105	171,009	0.83
2	Quarantine life	75,439	86,983	0.87
3	Solidarity	37,424	46,164	0.80
4	Call for action	29,134	31,809	0.92
5	Evidence and facts	27,957	31,819	0.88
6	Politics	21,724	24,346	0.89
7	Post-pandemic life	20,149	20,442	0.99
8	Shortage panic	15,255	16,815	0.91
9	Conflict	2095	3550	0.60

**Figure 2:**
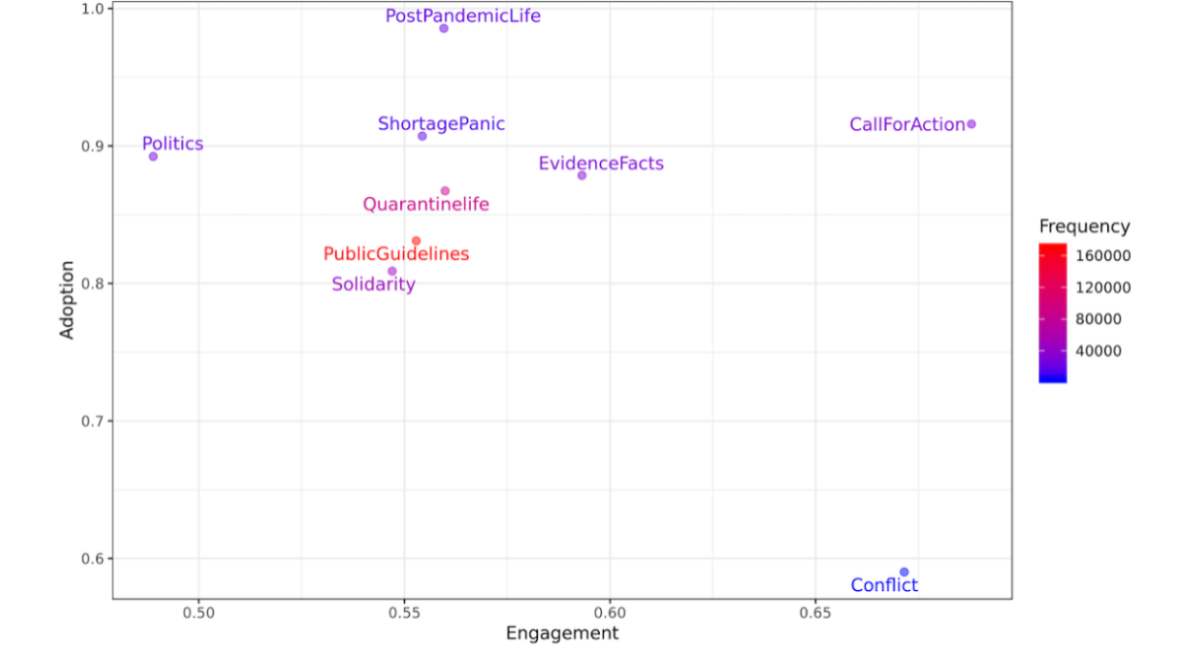
Distribution of the frames "public engagement" and "public adoption".

The distribution of public engagement and public adoption is illustrated in Figure 2. According to this figure, although conflict had the lowest frequency (n=3550), it received the second-highest engagement score (0.67). Public health guidelines had the highest frequency (n=171,009), followed by quarantine life (n=86,983); however, their public adoption and engagement scores were not high. Pearson correlation did not show any correlation between public adoption and public engagement: *r_8_*=–0.46, *P*=.18.

**Figure 3:**
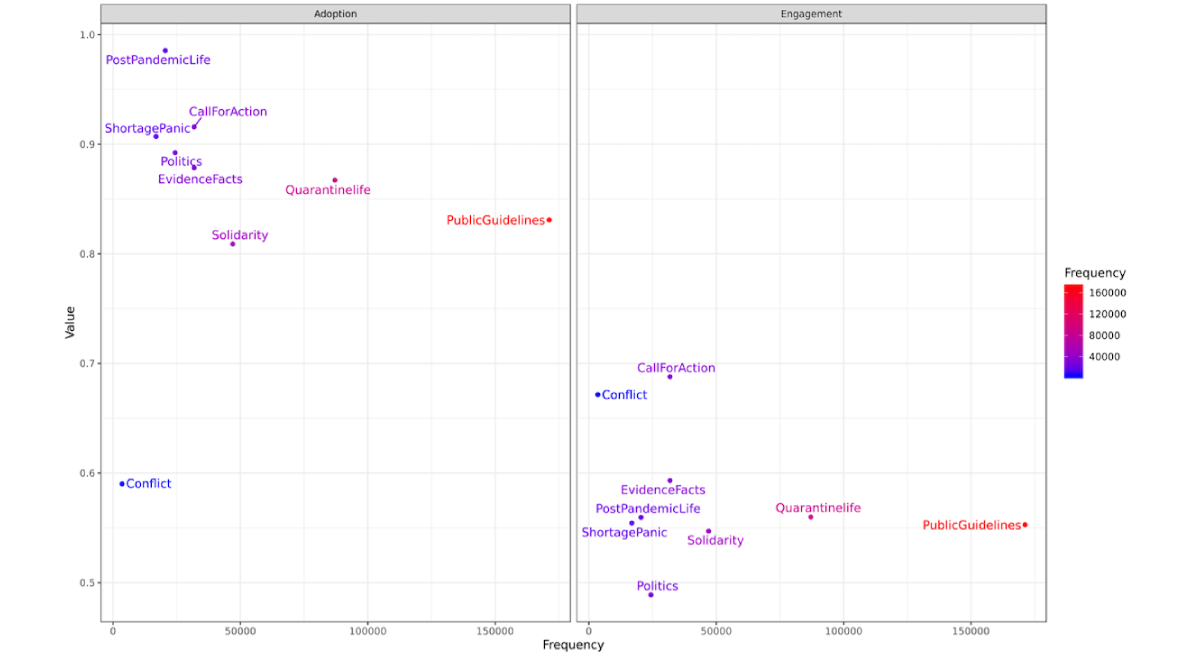
Distribution of the frames "public adoption" and "public engagement" in terms of frequency.

Figure 3 presents the distribution of public engagement with and public adoption of frames in terms of frame frequency. No meaningful patterns can be observed in frame engagement and adoption based on frequency. Pearson correlation showed no correlation between engagement and frequency (*r_8_*=–0.26, *P*=.46) nor between adoption and frequency (*r_8_*=0.13, *P*=.70). The insignificant results could mean that, if a frame receives high adoption by the people, it does not indicate that the frame would also achieve a high engagement (or vice versa).

## Discussion

### Principal Findings

This study analyzed the trending and associated hashtags in March 2020 during the initial phases of the COVID-19 pandemic. We found 9 frames in the public discourse on Twitter: “public health guidelines,” “quarantine life,” “solidarity,” “evidence and facts,” “call for action,” “politics,” “post-pandemic life,” “shortage panic,” and “conflict.” These frames and the hashtags within them such as #SupportSmallBusinesses and #StayPositive can be used to identify the types of information that should be delivered during any public health crisis, specifically the initial phase of the crisis. Furthermore, some of the frames such as solidarity, evidence and facts, call for action, politics, shortage panic, and conflict can be used in any local or global crisis such as social movements, earthquakes, tornadoes, hurricanes, and floods. For instance, the solidarity frame can be used to design messages to encourage people during an earthquake to show solidarity to those impacted by the crisis.

This study also evaluated public attention to the identified frames by assessing the amount of public engagement with and public adoption of frames. Among all frames, “call for action” had the highest engagement score, followed by “conflict” and “evidence and facts.” Additionally, “post-pandemic life” had the highest adoption score, followed by “call for action” and “shortage panic.”

According to Price and Tewksbury [59], framing is more effective when it is relevant to people, such as when the frames resonate with the audience’s beliefs or ideology [20]. The effects of frames on public opinion could be more substantial when the quality or logic of the argument and source credibility are more reliable [60]. The frames with greater public attention could be used strategically to design messages that affect public opinion more efficiently during public health emergencies. Additionally, these frames could be used to increase the performance of social media posts and encourage public compliance with public health messages.

### Theoretical Contributions and Implications

This study informs framing theory and research in several ways, mentioned in the following sections.

#### Frames as the Manifestation of COVID-19 Consequences

This research informs framing theory by indicating that frame analysis of public discourse on social media via hashtags is valuable not only in understanding public opinion about various aspects of the pandemic but also in recognizing the consequences of the pandemic on people’s lives, such as panic over the shortage of products.

The findings indicated that frames are the manifestation of COVID-19 consequences. The consequences discussed by people on Twitter about COVID-19 could be classified and analyzed based on whether (1) they were exclusively related to COVID-19, such as hand hygiene or isolation (ie, people are isolated or wash their hands because of COVID-19); (2) consequences could be related to any health crisis such as social support of vulnerable groups (ie, vulnerable groups require social support in any pandemic); and (3) consequences were generic irrespective of COVID-19, such as homeschooling or remote working (ie, some people homeschooled or worked remotely even before the pandemic).

#### Motivations and Sentiments in Frames

The findings showed that hashtags can promote intrinsic or extrinsic motivations among social media users. Hashtags can also convey a positive or negative sentiment. For instance, #StayHomeStayHealthy promotes an extrinsic motivation in people because this hashtag motivates people to stay home for external factors, such as prevention of contracting or spreading the virus. Another example could be #StayHomeStayHealthy, which has a positive sentiment because it indicates that staying healthy is a positive aspect of staying home.

#### Frames Promote the Collective Interest

Most hashtags promoted collective interest among people. For instance, #SocialDistancingNow was used by Twitter users to motivate people to prevent the potential transmission of COVID-19 for the public good. Another example could be #WearAMask in public, which promotes a collective interest to prevent the spread of the virus rather than merely preventing oneself from contracting the virus [61].

#### Conflict and Disagreement With Evidence and Facts

People used the “conflict” frame to discredit the evidence, facts, and updates about the COVID-19 virus, such as its fatality rates. Previous studies indicate that the news media sometimes use the “conflict” frame to report disagreements and arguments among people or groups to capture more audiences [42]. Using the conflict frame in social media posts can “increase the perceived seriousness and news value of an event” [62].

The conflict frame seems to be important in both the news media and public discourse during public health emergencies. Shih et al [14] stated that the conflict frame (defined as the arguments and disagreements among news sources) is the main frame in the media coverage of public health pandemics. Park et al [18] also found that conflict was a main frame in the Korean news media tweets about COVID-19. The popularity of the conflict frame demonstrates that there are always groups of people with disagreements about facts and evidence during public health emergencies.

This frame had the lowest frequency and lowest public adoption score among all frames identified in this study. A few previous studies have also indicated that news with conflict frames has been shared less on social media than news without conflict frames [62]. However, in this study, the conflict frame received the second-highest score for public engagement. This high score could be an indication that hashtags with conflicting information, such as #FilmYourHospital, are highly engaging on social media, despite their low frequency. A low adoption score with a high engagement score could indicate a focused community structure [51].

The “conflict” and “evidence and facts” frames indicate the presence of a chain of reactions, in that in response to the people who post information about facts and updates related to COVID-19 (ie, the evidence and facts frame), another group of people used the conflict frame to challenge those facts and updates (ie, the conflict frame). Some people would then, in turn, react to the conflict frame by sharing evidence and facts about the virus.

#### Vulnerable Groups

Our results indicated that frame analysis could be used to identify groups of vulnerable populations (eg, seniors, workers, and local and small businesses) and how the people frame their concerns and opinions about the needs of such populations on Twitter. It was found that vulnerable populations have been in need of prompt attention and support during the COVID-19 pandemic. For instance, seniors require social support, workers need safe workplaces, and local and small businesses need financial support. The findings did not show any hashtag referring to race or ethnic minority groups and health disparity populations, such as refugees, immigrants, homeless individuals, countries with fewer resources, or other marginalized communities. Overall, frame analysis through hashtags can be used to identify the populations that need more attention and support during global crises.

#### Accountability of Gatekeepers

Frame analysis can also identify who or which groups are responsible for managing a public crisis in the public view. The “call to action” frame indicated that Twitter users held federal and local governments accountable for their lack of actions regarding the closure of public places like beaches or restaurants. The “politics” frame also demonstrated people’s reaction to politicians’ decisions, policies, and insufficient actions during the pandemic. The politics frame includes hashtags that show those responsible for the situation in the public view. For instance, many hashtags in this category were used to make the US President accountable for the increasing number of COVID-19 cases and deaths. This frame could also show a lack of trust between politicians and the public, which often occurs during the initial phases of a pandemic [63].

#### The Use of Humor in Public Health Messages

The results in this study inform framing research by showing that people used hashtags such as #QuarantineCat or #HowToKeepPeopleHome to share information with humorous content. These hashtags were not directly related to COVID-19 but were generated and used by Twitter users due to COVID-19. Additionally, people used these hashtags to share their experiences of living in isolation. Some tweets with these hashtags were used to make fun out of the lived experience and actual difficulties people were dealing with during the pandemic. The use of humor in tweets has been previously reported in some past studies [64,65]. For instance, Kopper [65] noted that the use of humor in diplomatic tweets has a conflict-mediating role. In this study, the use of humor in public health tweets had a role in mitigating public anxiety or fear as the consequences of the pandemic. Perhaps people use humor in tweets to convey complex public health messages that are more attractive to audiences.

Although humorous hashtags may simply not seem appropriate for use in public health communication, they can make up part of a broader information-sharing strategy. For example, to communicate safety guidelines, social media posts could be labeled with humorous hashtags that are appealing. The safety-related messages may then be transferred to and adopted by the public through such hashtags. Additionally, practitioners, activists, organizations, and authorities can actively embed relevant and meaningful hashtags into their posts that are framed more generally within appealing concepts.

### Practical Implications

#### Designing Health Messages

The frames found in this study can also facilitate the communication on social media of various aspects of COVID-19 or any future health crisis. They can be used as a starting point for designing effective messages to address people’s needs and concerns during future public health crises [18]. For instance, based on the current research, it can be suggested that health messages should be communicated with empathy, as also proposed in the study by Hyland-Wood et al [66]. These frames can support the public in the expression of their feelings and opinions during global crises [21]. The frames can also be used to influence public opinion, behavior, and actions about critical and complex issues related to COVID-19 (see [33]).

Governments, practitioners, organizations, activists, and authorities can take advantage of hashtags to frame their messages to inform the public about the consequences of lack of adherence to public health guidelines. For instance, governments and public health officials need to share actionable guidelines with citizens during public crises and inform them about what is happening. These activities require understanding of how messages should be framed and presented so that risk communications are accepted and adopted by the public [67]. If health communication officers, political officials, and other authorities wish to encourage citizens to follow public health guidelines, stay optimistic, and be supportive during a health crisis, they can frame their messages using the hashtags identified in this research. For instance, a message by the WHO could be framed as follows: “#WeAreInThisTogether: #StayStrong at home and post a video or picture of your dogs or cats in #Quarantine. Use #QuarantineCats or #QuarantineDogs to help others see your posts.”

#### Issues and Their Roots

The results indicated that framing can also be used to identify the roots of the issues discussed by people on social media during public health crises. For instance, framing could be used to understand when, how, and about which topics false information is being distributed. The results indicated that #FilmYourHospital was a hashtag used by people to frame disagreements with evidence and facts about the COVID-19 pandemic. Once such problems and their roots are discovered through frame analysis, officials can find solutions, which, in this case, would be to neutralize and thwart false information dissemination.

Frame analysis can be used to identify what people are panicked about during a public health crisis. Another issue discussed by people was the “shortage panic,” referring to people’s reactions to the shortage and unavailability of products in the early days of the pandemic in March 2020. This panic could possibly be due to uncertainty in the public about what will happen in the future, lack of real-time communication with the public, or lack of trust between people and government officials. The study by Naeem [68] also indicated that uncertainty about COVID-19 triggered people to buy extra necessary food items, which resulted in the buying panic during the COVID-19 pandemic.

### Methodological Contributions

This research contributes to the methodology of framing research on social media in several ways, as explained in the following sections.

#### Accuracy in Data Collection

In this research, the trending and associated hashtags were used to collect tweets. Using hashtags can increase the accuracy of collecting relevant tweets. The hashtag search approach is more accurate than the keyword-based search approach used in some past studies, such as that by Jang and Hart [69], mainly because people use hashtags to describe their tweets [70]. Jang and Hart [69] searched “climate change AND real” to find tweets associated with the “real frames” or searched “climate change AND action” to find tweets about “action frames.” A major issue with the keyword-based search and analysis is that it may result in retrieving extraneous tweets or excluding relevant ones, as stated by Jang and Hart [69].

#### Using Trending Hashtags to Identify Frames

Unlike most previous framing studies on Twitter that have solely analyzed tweets to identify frames, this study analyzed trending and associated hashtags along with the tweets containing those hashtags to identify frames. Jang and Hart [69] stated that an effective approach for analyzing frames is “to identify unique components of public rhetoric that clearly represent single frames of a more complex issue.” Hashtags are unique components that represent frames of complex issues on social media. Moy and Bosch [30] noted that frames provide “meaning about social phenomena through the highlighting and packaging of information.” Hashtags can also be used to give meaning to and highlight social phenomena. In the Literature Review section, the rationale for using (trending) hashtags for identifying frames was discussed.

#### Measuring Public Attention on Twitter

There is no standard way for measuring public attention on social media [47]. This study contributes to the methodology of framing research by examining the amount of public attention to frames by evaluating public adoption of frames and public engagement with frames [51]. Rarely any previous framing research has used this approach to measure the effect of frames on public attention on social media. This approach can also be used to identify the framing effect of the news media on public attention on social media in future studies.

### Study Limitations

The study limitations were mostly related to data collection, some of which were beyond our control. For instance, due to the Twitter API restrictions in collecting historical data, all tweets associated with the trending and associated hashtags could not be collected. We tried to mitigate this limitation by using 2 other software for collecting historical data, as explained in the Data Collection section.

Another limitation is that sampling tweets based on trending hashtags may not represent all Twitter communication. Hashtags may only represent a specific subset of Twitter communication. All Twitter users do not use hashtags in their online communications on social media [71,72].

As mentioned, to identify the frames, the trending hashtags on Twitter in March 2020 were collected. Collecting data in other months may lead to identifying different frames.

Additionally, this research may have missed collecting all trending hashtags about COVID-19 in March 2020 for several reasons. First, Twitter determines emerging and trending topics (topics that are popular now) based on who the users (researchers who collect the data) follow, their interests, their locations, and other criteria (see Twitter trends FAQs). Second, a hashtag may be trending for a couple of hours, a day, or in rare cases, more than a day. A hashtag that is trending today may not be trending tomorrow, but people may still use the hashtag for a short or long period of time. The hashtag may disappear temporarily after a few days, may gain attention again in the future, or may disappear permanently [36].

### Future Research

This study answers important questions about the frames in public discourse during the initial phase of the COVID-19 pandemic, but also raises questions to investigate in future research. This research identified 9 frames, which provide useful orientation for future empirical and theoretical research that aims to investigate the frames on social media during global (health) crises. Future studies can expand these frames and apply them to other global crises.

This study did not investigate how different groups of people that create the public, like citizens, officials, researchers, journalists, and organizations, frame COVID-19 differently, which is worth studying in future studies.

Experimental designs should be designed to manipulate the type and category of frames (while keeping other variables constant) to understand to what degree hashtags affect public collective attention (see [73]) on social media.

Future research could also investigate (1) how frames and hashtags emerge, evolve, and operate and (2) how they succeed in achieving sustainability during different phases of a pandemic, such as (1) precrisis, (2) the initial event, (3) maintenance, (4) resolution, and (5) evolution [74].

Additionally, public compliance to health messages and guidelines is different than public attention to frames. Public compliance to COVID-19 can be evaluated by different measures such as “compliance to public health and social measures in preventing the spread of COVID-19” [75]. Future research should investigate how health messages should be designed and framed to increase public compliance to health guidelines during pandemics.

### Conclusion

This is one of the first studies to use trending hashtags to analyze and identify frames on social media. It contributes to framing theory and research by showing that frames represent the consequences of a public health emergency such as COVID-19. Additionally, the findings inform framing theory and research by showing that the methodological advantages of using trending and associated hashtags lie not only in their ability to understanding the frames that are the focal point of the public but also in their potential to allow researchers to measure public attention to those frames. The results indicated that some frames are more appealing during a global pandemic than others, such as “call for action,” therefore receiving greater public adoption and engagement. It was found that the frequency of a frame on social media does not necessarily mean greater public adoption of or engagement with the frame.

## Appendix

Multimedia Appendix 1 Framing of COVID-19 by the news media.

Multimedia Appendix 2 Framing of the COVID-19 pandemic in the public discourse on Twitter.

## Abbreviations

API: application programming interface
WHO: World Health Organization
