# Supplementary material for: Framing of and Attention to COVID-19 on Twitter: Thematic Analysis of Hashtags
Source: J Med Internet Res. 2021 Sep 10;23(9):e30800. doi: 10.2196/30800 (PMC8437404; doi:10.2196/30800)
Supplement: Multimedia Appendix 1 [file jmir_v23i9e30800_app1.docx]

Framing of COVID-19 by the news media

| # | Study population | Key findings of past research | Source |
| --- | --- | --- | --- |
| 1 | Global media outlets, such as *BBC*, *News York Times, CNN*, *and People’s Daily.* | (a) Human interest: refers to framing massages in a way to personalize and dramatize news to attract greater audience interest (Semetko & Valkenburg, 2000), (b) Fear/scaremongering: framing messages in an exaggerated way to cause fear or panic in the public (Ogbodo et al., 2020). | Ogbodo et al. (2020) |
| 2 | Front pages of 12 well-known Canadian news media sources | (a) Health crisis: the most dominant frame, (b) Chinese outbreak, (c) Economic crisis, (d) Helping Canadians, (e) Social impact, (f) Western deterioration (Poirier et al., 2020). | Poirier et al. (2020) |
| 3 | Tweets of Korean news media | Medical frames: health issues related to COVID-19.  Non-medical frames: (a) Conflict: disagreements about COVID-19, (b) Human interest: the positive role of people in handling COVID-19, (c) Attribution of responsibility: government and public officials are responsible for the crisis, (d) Morality: moral and ethical messages (Park et al., 2020). | Park et al. (2020) |
| 4 | Spanish newspapers | (a) Livelihood: family life and children, (b) Public health professional: news about the department of public health, (c) Pandemic update: contagion and death poll, (d) Politics: general political news, (e) State of alarm: government’s announcement and policy update, (f) Economy: the effect of the pandemic on the Spanish economy, (g) COVID information: general information about the pandemic, (h) Lockdown, (i) Hospital (Yu et al., 2020, p. 4). | Yu et al. (2020) |
