# Supplementary material for: Framing of and Attention to COVID-19 on Twitter: Thematic Analysis of Hashtags
Source: J Med Internet Res. 2021 Sep 10;23(9):e30800. doi: 10.2196/30800 (PMC8437404; doi:10.2196/30800)
Supplement: Multimedia Appendix 2 [file jmir_v23i9e30800_app2.docx]

Framing of the COVID-19 pandemic in public discourse on Twitter

| No | Hashtags^a^ as the building blocks of frames (First phase of thematic analysis) | Descriptions of tweets containing hashtags  (Second phase of thematic analysis) | Themes representing the consequences of COVID-19  (Third and fourth phases of thematic analysis) | Frames  (Fifth phase of thematic analysis) |
| --- | --- | --- | --- | --- |
| 1 | #StayHome*, #StayHomeSaveLives*, #StayAtHome*, #StayHomeStaySafe, #StayHomeStayStrong, #StayHomeStayHealthy, #StayInside  #SaferAtHome, #SelfQuarantine, #StayHomeStaySafe, #SelfIsolation, #SelfIsolating | Encouraging and advising people to stay home to prevent from spreading of the virus. | Isolation | Public health guidelines |
|  | #HowtoKeepPeopleHome* | Encouraging people to stay home. This hashtag is sometimes used in humorous messages to encourage people stay home. |  |  |
|  | #SocialDistancing*, #KeepYourDistance  #PhysicalDistancing, #SocialDistance, #SocialDistancingNow, #YouAreTooCloseIf* | Practicing social distancing. These hashtags are used by some people to show how they are practicing social distancing. Some hashtags like #YouAreTooCloseIf are used in humorous messages about social distancing and its importance. | Social distancing |  |
|  | #DontBeASpreader (trending hashtag), #StoptheSpread | Asking people to practice social distancing and other preventative measures to stop the spread of the virus. |  |  |
|  | #WashYourHands*, #HandWashing*  #SafeHand*, #SafeHands*, #HandWashChallenge* | These hashtags are used to show the importance of hand hygiene, encourage and challenge people to wash their hands, and show how hands should be washed. | Hand hygiene |  |
|  | #HandSanitizers | This hashtag is used to refer to the existence of hand sanitizers in some stores; donation of hand sanitizers; home-made hand sanitizers; and shortage of hand sanitizers. |  |  |
|  | #FaceMask, #WearAMask, #DontTouchYourFace | Asking people not to touch their faces to avoid contracting the virus. | Face hygiene |  |
|  | #CoronaTips*, #CoronaVirusTips*, #CoronaVirusPrevention*, #CoronaVirusAwareness* | Providing tips and guidelines, and knowledge about various aspects of the virus. | Preventive tips and awareness |  |
| 2 | #HomeOffice*, #WorkingFromHome  #WorkFromHome | These hashtags were used to share pictures or videos of home offices and people working from home. | Home as office | Quarantine life |
|  | #HomeSchooling*, #HomeSchool* | These hashtags were used to share pictures or videos of kids doing their school activities from home. | Home schooling |  |
|  | #QuarantineLife (trending hashtag), #Day38OfLockdown, #QuarantineActivities, #QuarantineAndChill | Personal life during the quarantine. These hashtags were used to show daily life during the quarantine. | Life in quarantine |  |
|  | #After3WeeksWithMyFamily (trending hashtag), #SideEffectsofQuarantineLife (trending hashtag), #StayHomeChallenge* | Tweets with a sense of humor displaying what people do and how they feel during the quarantine. |  |  |
|  | #QuarantineCats*, #QuarantineDogs* | People used these hashtags to share tweets, often with humorous content, about their pets during quarantine. | Pets and quarantine |  |
|  | #LockDown, #CoronaLockDown, #CoronaVirusLockDown, #ThelockDown | These hashtags represent various aspects of daily life during the lockdown. | Lockdown |  |
|  | #OnlineLearning, #RemoteLearning, #DistanceLearning, #RemoteWorking | Impact of the pandemic on how people study, learn, and work during the quarantine. | Remote Working/Learning |  |
| 3 | #StayStrong*, #StayPositive*, #WeAreInThisTogether, #StayHealthy, #StrongerTogether, #TogetherWeCan, #StayConnected | Inspiring, encouraging, giving hope, and supporting each other as part of a solution to the pandemic. | Social support | Solidarity |
|  | #WeServeAndProtect | This hashtag was used to show voluntary services (such as disinfecting facilities) during the pandemic. | Voluntary service to communities |  |
|  | #CoronaWarriors, #ClapForCarers  #HealthCareHeroes, #ClapForOurCarers | Showing appreciation for the frontline workers in fighting the pandemic, specifically health care professionals. | Acknowledging health care professionals |  |
|  | #TogetherApart (trending hashtag), #AloneTogether, #PlayApartTogether | Emphasizing that best way to be together is to stay apart until the pandemic is over. | Showing unity |  |
|  | #ArmyMissesBTS* | Solidarity for canceled concert of the BTS or Bangtan Boys (a seven-member South Korean boy band) because of the pandemic. | Solidarity with canceled events |  |
|  | #OpenforTakeout*, #OpenForDelivery (trending hashtag), #SupportSmallBusiness  #SmallBusiness, #SupportLocal | Supporting small and local businesses and locally owned restaurants that are open for takeout or delivery. | Supporting local and small business |  |
|  | #ProtectOurSeniors^*^*, #ProtectSeniors*, #ProtectTheSeniors*, #ProtectTheVulnerable, #ProtectHealthCareWorkers, #ProtecTheAllCareWorkers | Supporting vulnerable groups, such as seniors, and those who are in the frontline of fighting the pandemic, such as healthcare workers. | Supporting vulnerable groups |  |
|  | #AmazonStrike (trending hashtag), #InstaCartStrike*, #ProtectAmazonWorkers, #EssentialWorkers | Supporting workers and the unsafe working environments during the pandemic. | Supporting workers |  |
| 4 | #CloseTheSchools (trending hashtag), #CloseTheSchoolsNow*, #SchoolClosure*, #CloseTheLibraries*, #CloseTheLibrary*, #CloseTheColleges*, #CloseTheUniversities, #CloseTheCamps*, #CloseTheDayCare*, #Covid19Walkout, #CloseTheBeaches (trending hashtag), #CloseThe*, #CloseThePubs*, #CloseTheBars*, #ShutItDown | Asking federal and local governments to shut down public places, like beaches and schools. | Appealing to the authorities to close public places | Call for action |
|  | #FlattenTheCurve | Encouraging people to play their own part in flattening the curve. | Collective responsibility and action |  |
| 5 | #CoronaVirusUpdates (trending hashtag), #CoronaUpdate* | Updates about various aspects of the virus like the total number of deaths or new cases, or updates on new evidence about the virus. | Updates on the pandemic | Evidence and facts |
|  | #CoronaVirusTruth | The hashtag was used to bring public attention to the facts about COVID-19, such as the increasing number of deaths in some countries like USA. | Facts about the virus |  |
| 6 | #TrumpLiedPeopleDied (trending hashtag), #ChinaLiedPeopleDied (trending hashtag), #YangWasRight*, #FoxNewsLiedAsPeopleDied*, #TrumpPandemic*, #TrumpPlague, #TrumpLiesPeopleDie, #TrumpVirusCoverUp, #TrumpLiesAboutCoronavirus, #TrumpVirus | Making political officials (or news media) responsible for their actions, decisions, and policies. | Accountability of (political) gatekeepers | Politics |
| 7 | #WhenCoronaVirusIsOver (trending hashtag), #WhenILeaveMyHouseAgain (trending hashtag) | Future life and plans when the pandemic are over. Referring to how the world will change or how people will feel when the pandemic is over. These hashtags sometimes are used in tweets with humorous contents. | Life, feelings, and plans after the pandemic. | Post-pandemic life |
| 8 | #Panicbuying (trending hashtag), #ToiletPaper*, #ToiletPaperPanic (trending hashtag), #ToiletPaperCrisis*, #ToiletPaper*, #PanicShopping*, #CoronaPanic*, #ToiletPaperShortage*, #ToiletPaperEmergency*, #ToiletPaperApocalypse*, #ToiletPaperWars | People’s reactions in the preliminary phase of the pandemic, such as panic and anxiety to shortage of products (e.g., toiled papers or bottled water). | Toilet paper panic | Shortage panic |
| 9 | #FilmYourHospital (trending hashtag), #EmptyHospitals*, #FakeNews | Arguments and disagreements with information, news, and facts related to COVID-19. | Challenging the facts and updates | Conflict |

a The hashtags which were found by searching in the search box of the Twitter website (i.e., hashtags associated with trending hashtags) are labeled by the asterisk sign (*). Although, trending and associated hashtags were the main building blocks of identifying frames, other hashtags in the dataset that were relevant to at least one of the frame categories were also added to the table to enrich the list of hashtags in each category (i.e., the hashtags without the asterisk sign).
